# Volatile Flavoromics of Four *Mesona chinensis* Benth Cultivars: Metabolomic Basis for the Superior Aroma of the Zengcheng Elite Cultivar

**DOI:** 10.3390/ijms26178713

**Published:** 2025-09-07

**Authors:** Yuqing Niu, Yujing Zhu, Meixia Zheng, Yanming Zhu, Hong Chen, Dagang Tian, Hailan Su

**Affiliations:** 1 Institute of Crop Sciences, Fujian Academy of Agricultural Sciences, Fuzhou 350013, China; niuyuqing804@163.com (Y.N.); zyjingfz@163.com (Y.Z.); zhengmeixia2005@163.com (M.Z.); mumuzym@163.com (Y.Z.); chenhong6313@163.com (H.C.); 2 Biotechnology Research Institute, Fujian Academy of Agricultural Sciences, Fuzhou 350013, China

**Keywords:** *Mesona chinensis* Benth, targeted metabolomics techniques, flavor quality formation, Zengcheng

## Abstract

Zengcheng (ZC), a superior cultivar of *Mesona chinensis* Benth, is distinguished by its unique sensory attributes and exceptional phytochemical quality, rendering it the cultivar of choice for commercial production. In this investigation, we employed comprehensive targeted metabolomics to systematically delineate the volatile metabolome of ZC in comparison to three relevant counterparts grown under uniform field conditions and harvested at the same developmental stage. Our primary objectives were to (i) quantitatively characterize the metabolomic divergence between ZC and the comparative cultivars, and (ii) elucidate the molecular basis underlying ZC’s characteristic flavor signature. Through high-throughput gas chromatography–mass spectrometry (GC-MS) analysis, a total of 1767 volatile metabolites were confidently annotated, with terpenoids and esters identified as the predominant chemical classes. Notably, ZC exhibited significantly elevated levels of terpenoids, esters, and aldehydes, which collectively conferred an intensified green, sweet, herbal, and fruity aroma. Key discriminatory metabolites included geranyl formate and geranic acid (imparting a pronounced green note), which were identified as pivotal determinants of ZC’s unique flavor profile. These findings provide mechanistic insights into the biochemical pathways governing flavor development in *M. chinensis* Benth and establish a robust foundation for precision-breeding strategies aimed at enhancing sensory quality and developing elite cultivars.

## 1. Introduction

In recent years, the vast biodiversity of the plant kingdom has garnered special interest as a source of specific secondary metabolites, which are pivotal not only for plant defense and stress adaptation but also for their diverse beneficial effects on human health, including antioxidant, anti-inflammatory, and cardioprotective activities [1,2,3]. *Mesona chinensis* Benth is an important medicinal and edible plant resource in China and Southeast Asia, which has high nutritional value and the effect of relieving summer heat and thirst [4]. *M. chinensis* Benth is abundant in polysaccharides, flavonoids, terpenoids, and phenolic compounds, and exhibits antioxidant properties, modulates intestinal flora, and demonstrates hypoglycemic, antibacterial, and antiviral activities [5,6,7,8]. It is often used to make summer-relieving drinks. Its function of treating thirst and hypertension has been recorded in Chinese Materia Medica and the Dictionary of Chinese Medicine. With the development of the herbal tea industry, its demand is increasing, and the planting scale of *M. chinensis* Benth is gradually expanding. In terms of dry weight, the annual production of *M. chinensis* Benth is about 4500–10,000 tons (fresh weight is about 24,000–60,000 tons). Nonetheless, there exists a scarcity of *M. chinensis* Benth cultivars that are appropriate for the production of herbal tea. Among them, the Zengcheng (ZC) cultivar commands the largest market share because of its mellow, refreshing infusion with low bitterness—traits that align closely with consumer preference. Consequently, flavor has emerged as a key breeding target for *M. chinensis* Benth.

Volatile organic compounds (VOCs) are major determinants of plant-derived flavor. For example, GC–MS identified 46 components in Zhoupigan, which defined Zhoupigan’s distinct aroma [9]. There were 544 VOCs in citrus fruits at different growth stages, among which 91 were terpenoids [10]. A total of 755 volatile metabolites were identified, which are associated with the taste, aroma, color, and quality of Ziziphus jujube leaf black tea [11]. The metabolites present in various plants, as well as in different cultivars of the same species, exhibit significant variability. *M. chinensis* Benth contains a diverse array of both volatile and non-volatile metabolites, which influence consumer acceptability. While the volatile profiles of many medicinal and edible plants have been characterized, the flavor chemistry of *M. chinensis* Benth remains notably underexplored. Previous studies have primarily focused on the health benefits of its non-volatile bioactive compounds, such as polysaccharides and flavonoids [4,5,6,7,8]. Few investigations have systematically addressed its volatile aroma compounds. Existing reports are often limited to identifying a small number of major constituents without linking them to sensory attributes or cultivar differentiation [12]. This lack of a comprehensive, cultivar-specific volatilome map hinders the understanding of the biochemical basis of its prized aroma and restricts targeted breeding efforts for improved flavor quality. A systematic comparative metabolomics survey across cultivars is therefore imperative for identifying flavor-controlling metabolites and for guiding precision-breeding strategies.

Advances in high-throughput, broad-target GC–MS metabolomics now enable comprehensive profiling of plant volatilomes with high precision and wide dynamic range [13,14], as demonstrated in recent methodological advancements [15]. Integration of these data with multivariate statistics (principal component analysis (PCA)), orthogonal partial least squares discriminant analysis (OPLS-DA), hierarchical cluster analysis (HCA), and pathway mapping (KEGG) provides a powerful framework for dissecting biosynthetic routes and regulatory nodes underlying flavor chemistry. Here, we apply this integrated approach to contrast the volatile metabolomes of ZC and three commercially relevant cultivars. By quantifying differentially accumulated metabolites (DAMs) and mapping them to sensory descriptors, we elucidate the biochemical mechanisms that confer ZC’s distinctive flavor. These findings not only establish a scientific basis for flavor-oriented breeding, but also open avenues for the discovery of novel bioactive constituents for functional foods and pharmaceutical applications.

## 2. Results

### 2.1. Volatile Metabolite Profiling of Four M. chinensis Benth Cultivars

To comprehensively characterize the metabolic profiles of four cultivars of *M. chinensis* Benth, we applied broad-target GC–MS metabolomics to leaf samples of four *M. chinensis* Benth cultivars (Figure S1). A total of 1767 volatile metabolites were identified and classified into 15 categories: acids (4.93%), alcohols (8.55%), aldehydes (5.89%), amines (3.51%), aromatic compounds (2.43%), esters (18.52%), ethers (2.1%), halogenated hydrocarbons (0.85%), heterocyclic compounds (10.19%), hydrocarbons (5.04%), ketones (12.29%), nitrogen compounds (1.13%), phenols (4.25%), sulfur compounds (0.45%), and terpenoids (19.88%). Notably, terpenoids and esters collectively accounted for 38.40% of the total metabolites.

**Figure 1:**
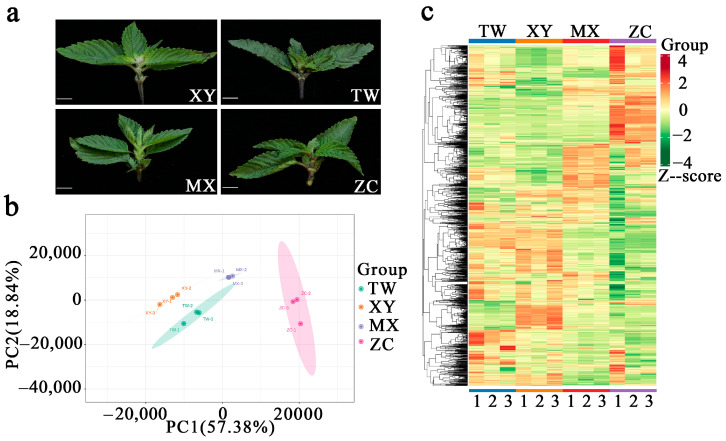
Metabolite profiles of four *M. chinensis* Benth cultivars. (**a**) Photographs of the *M. chinensis* Benth used in this study (scale bar = 1 cm). (**b**) Principal component analysis of metabolic profiles of four *M. chinensis* Benth cultivars. (**c**) Hierarchical clustering analysis was conducted on four *M. chinensis* Benth cultivars. In the resulting heatmap, each column corresponds to a specific provenance, while each row represents an individual metabolite. Metabolite abundance is visually represented by color, with red indicating relatively high abundance and green indicating relatively low abundance.

Unsupervised multivariate analysis was subsequently performed to dissect inter-cultivar metabolic divergence. Principal component analysis (PCA) and hierarchical clustering analysis (HCA) were conducted to elucidate metabolic differences among the four cultivars. PCA revealed distinct separation between cultivars (Figure 1b), with “ZC” exhibiting the most pronounced divergence, suggesting its distant phylogenetic relationship to others. The first two principal components explained 57.38% and 18.84% of metabolic variance, respectively. Low intra-variety variability in biological replicates indicated high experimental reproducibility. Consistent with PCA, HCA-based clustering heatmaps demonstrated significant divergence in metabolite accumulation patterns, particularly for ZC (Figure 1c). Analysis of volatile component distribution (Figure S2) showed distinct compositional hierarchies: Taiwan (TW): Terpenoids > Hydrocarbons > Esters; Xiaoye (XY): Terpenoids > Ketones > Esters; Minxuan (MX): Terpenoids > Esters > Ketones; ZC: Terpenoids > Aldehydes > Esters.

### 2.2. OPLS-DA and K-Means Reveal ZC-Specific Volatile Metabolome

**Figure 2:**
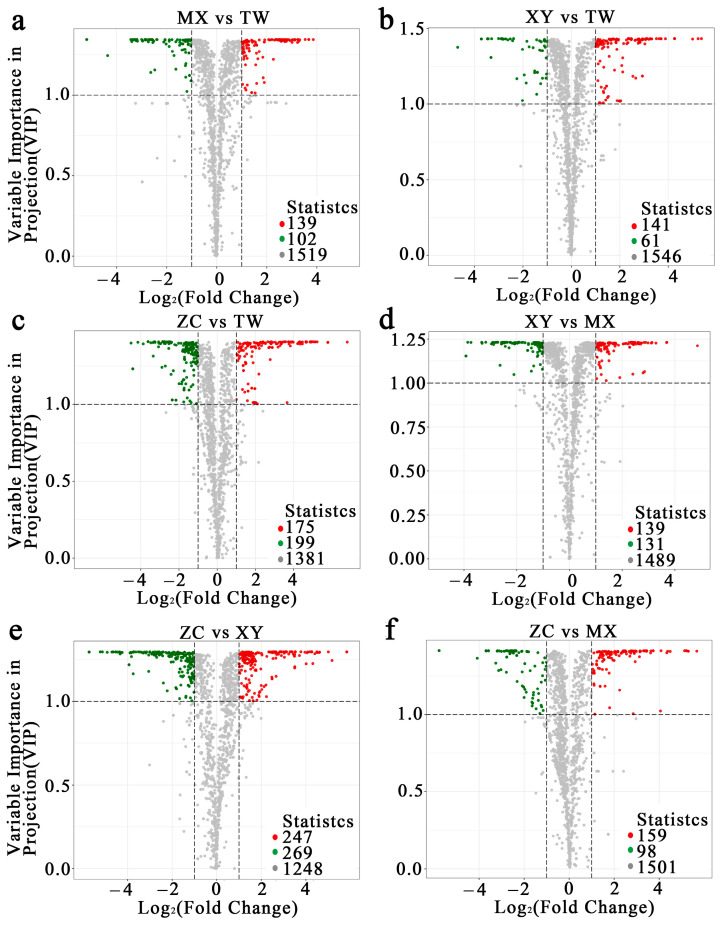
Volcanic map of differential metabolites. (**a**–**f**) In the volcano plot, each point corresponds to a metabolite, with green points indicating downregulated differential metabolites, red points indicating upregulated differential metabolites, and gray points indicating metabolites that are detected but do not exhibit significant differences. The horizontal axis represents the logarithmic value (log_2_FC) of the fold change in the relative abundance of a metabolite between the two sample groups. The vertical axis denotes the VIP value, where a higher value indicates a more significant difference and enhances the reliability of the screened differential metabolites.

**Figure 3:**
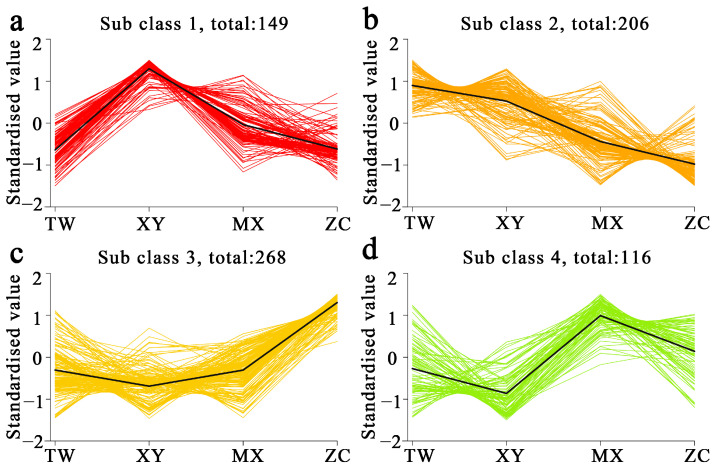
K-means clustering analysis was conducted on the differentially accumulated metabolites of four *M. chinensis* Benth cultivars. (**a**–**d**) The *y*-axis represents the standardized quantities of each metabolite, while the *x*-axis denotes the various samples. Black line: cluster center line, representing the average trend of all metabolites within the cluster.

To maximize cultivar discrimination, supervised OPLS-DA was performed (Figure S3). All models exhibited robust statistics, confirming clear metabolic separation in every pairwise comparison. Critically, the ZC cultivar consistently exhibited the most distinct metabolic signature, as evidenced by the highest number of differentially expressed metabolites (DEMs) in all comparisons where it was involved. DEMs were extracted from the corresponding S-plots using dual thresholds of VIP > 1 and |FC| ≥ 2. Across the six contrasts (Figure 2), the number of DEMs varied substantially among comparisons: the ZC vs. XY contrast exhibited the greatest divergence with 516 DEMs (247 upregulated, 269 downregulated in ZC), followed by ZC vs. TW (374 DEMs) and ZC vs. MX (257 DEMs). Comparisons among the other three cultivars yielded fewer DEMs, ranging from 202 (XY vs. TW) to 270 (XY vs. MX). ZC consistently displayed the largest DEM complement. This pattern unequivocally identifies ZC as the most metabolically distinct cultivar among the four. Subsequent K-means clustering (k = 4) of the 739 DEMs—after unit-variance scaling—produced four distinct abundance profiles (Figure 3): a total of 149 metabolites peaked in XY, 206 in TW, 268 in ZC, and 116 in MX. Over 60% of these DEMs were terpenoids, esters, or ketones, underscoring these pathways as primary drivers of volatile aroma divergence among the four cultivars. The significant enrichment of these particular compound classes in ZC provides the direct metabolic basis for its superior and complex flavor profile.

### 2.3. KEGG Pathway Enrichment Implicates Monoterpenoid Biosynthesis in the Distinctive Volatile Signature of ZC

**Figure 4:**
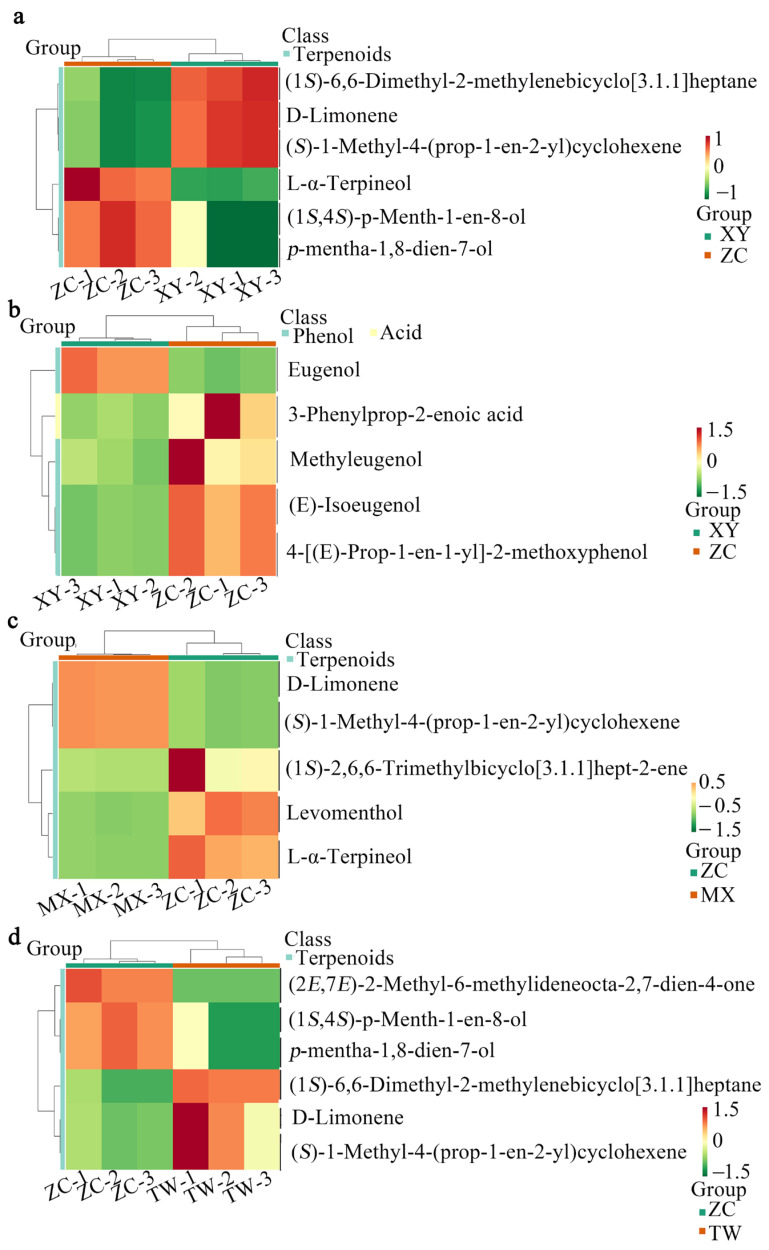
Cluster analysis of KEGG pathway differential metabolites. (**a**–**d**) The horizontal axis shows sample names, while the vertical axis displays differential metabolites. Colors indicate standardized relative content values, with red for high and green for low. The note bar above the heatmap indicates sample groups, and the tree diagram on the left shows hierarchical clustering of metabolites. The comment bar on the right of the cluster tree denotes substance classes, with different colors representing each class.

To delineate the biochemical basis of ZC’s unique chemotype, DEMs from ZC versus each of the other three cultivars were subjected to KEGG pathway enrichment. Statistically significant enrichment (adjusted *p* < 0.05) was consistently observed for terpenoid biosynthesis across all three contrasts—ZC vs. XY, ZC vs. MX, and ZC vs. TW (Figure 4a,c,d). In the ZC vs. XY comparison, phenylpropanoid and organic-acid metabolic pathways were additionally enriched (Figure 4b). Core metabolites driving these enrichments included D-limonene, (*S*)-1-Methyl-4-(prop-1-en-2-yl)cyclohexene, and L-α-terpineol, all of which map to the monoterpenoid biosynthetic pathway and collectively underpin the distinctive volatile signature of ZC. A detailed list of all metabolites, including their classifications and relative abundances, is provided in Supplementary Table S1.

### 2.4. Sensory-Omics Atlas: Metabolite Drivers of ZC’s Distinctive Flavor Signature

**Figure 5:**
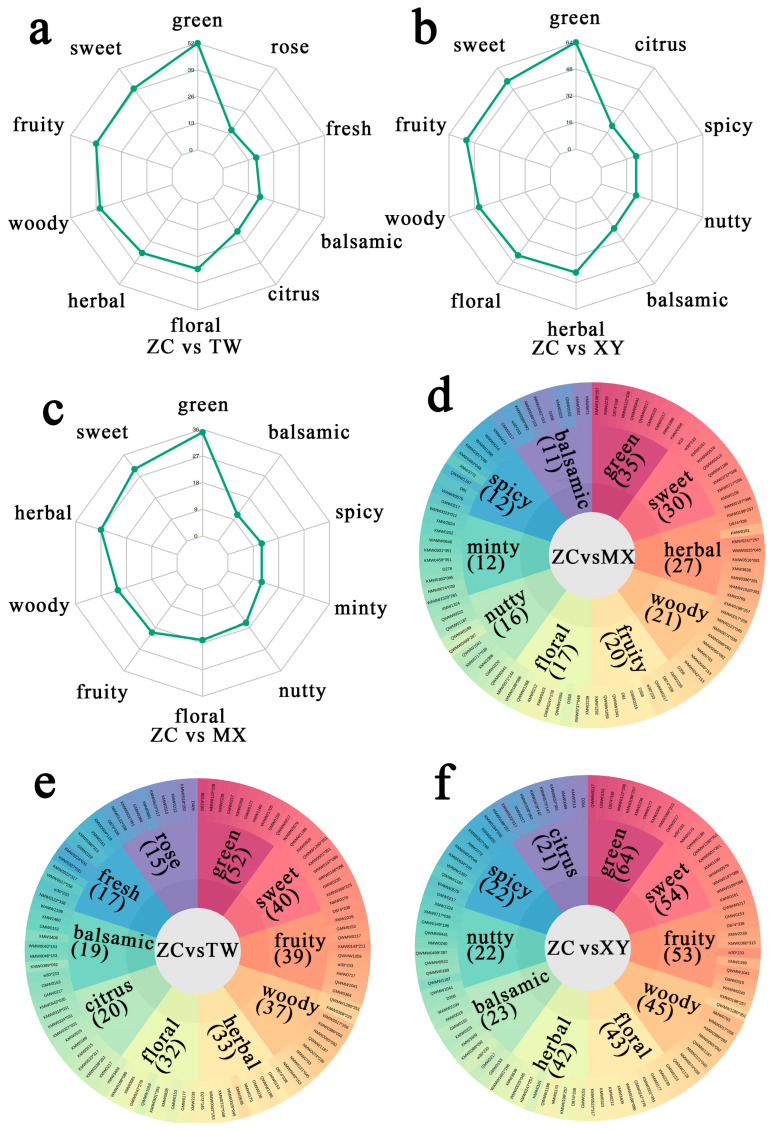
Radar map and differential metabolite flavor wheel for the analysis of differential sensory flavor characteristics of metabolites. (**a**–**c**) The outermost label represents a sensory flavor attribute, while the numeral associated with the green dot denotes the quantity of differential metabolites identified in relation to that specific sensory flavor attribute. (**d**–**f**) The innermost circle represents the comparison group, while the second circle shows the top 10 sensory flavor features with the most differential metabolites annotated (numbers in parentheses indicate the count).

Aroma-active volatiles were interrogated as quantitative proxies for sensory perception. DEMs from each pairwise comparison (ZC vs. MX, ZC vs. TW, ZC vs. XY) were mapped to curated sensory descriptors, and the ten most frequently annotated attributes were visualized via radar plots (Figure 5a–c) and integrated into a differential-metabolite flavor wheel (Figure 5d–f).

Our analysis identified several key differential compounds that are significantly more abundant in ZC and are directly responsible for its desirable aroma profile. Green note: ZC displayed the strongest green signature, supported by 35, 52, and 64 exclusive metabolites relative to MX, TW, and XY, respectively. Key contributors include geranic acid, geranyl formate, dimethyl butanedioate, β-methyl-benzeneethanol, and isoneral. Sweet note: Sweet perception was markedly elevated in ZC, driven by 30 (vs. MX), 40 (vs. TW), and 54 (vs. XY) metabolites, notably 3-Methylbutyl propanoate, 3-octanone, 2-Methylacetophenone, 1-octen-3-ol, and 5-methyl-2-acetylthiophene. Herbal note: A pronounced herbal character distinguished ZC, attributed to 27 (vs. MX), 33 (vs. TW), and 42 (vs. XY) metabolites, among which *4*-*tert*-butylcyclohexyl acetate, fenchone, 2,3-octanedione, piperitenone oxide, and (*R*)-2-methyl-5-(prop-1-en-2-yl)cyclohexanone are prominent. Fruity note: Fruitiness was significantly amplified in ZC via 20 (vs. MX), 39 (vs. TW), and 53 (vs. XY) metabolites, with 3-Methylbutyl propanoate, 1-octen-3-ol, 3-Methylbut-3-en-1-yl acetate, triacetin, and (*E*)-2-Methylpent-2-enoic acid as the dominant drivers.

Collectively, these quantitatively resolved metabolite–sensory linkages provide a molecular blueprint for the superior flavor complexity of ZC. The heightened abundance of key metabolites such as geranyl formate, geranic acid, and fenchone serves as a definitive chemical signature of the ZC cultivar and offers precise targets for aroma-oriented breeding and post-harvest processing innovations.

## 3. Discussion

Utilizing advanced targeted metabolomics technology, we quantitatively compared the volatile chemotypes of ZC and three additional *M. chinensis* Benth cultivars to decode the molecular determinants of their divergent flavor profiles. High-throughput detection methods, specifically the SPME Arrow extractor and SIM detection modes, were employed to elucidate the correlation between metabolite composition and the *M. chinensis* Benth’s flavor quality with enhanced sensitivity [16,17,18]. This study independently established a specific database of samples based on multiple species, literature, and some standard products and retention indices, including definite RT and qualitative and quantitative ions to select ion detection modes for precise scanning. The method is readily transferable to the volatolomic analysis of other medicinal or edible plants [16,17].

Consistent with reports in citrus [19], tea [20], and apple [21], terpenoids (19.88%) and esters (18.52%) dominated the *M. chinensis* Benth volatilome. ZC exhibited pronounced shifts in these classes, accumulating >60% of its DEMs among terpenoids, esters, and ketones that collectively translate into its signature green, sweet, herbal, woody, fruity, and floral notes. KEGG pathway enrichment underscored monoterpenoid biosynthesis as a central axis of divergence, plausibly controlled by enzymes such as limonene synthase. Concurrent enrichment of aldehydes (e.g., hexanal) in ZC suggests enhanced lipid-oxidative generation of grassy aromas, a mechanism previously documented in tea [20,22].

This study further identified that geranyl formate and geranic acid contributed to a more pronounced green flavor in ZC compared to other cultivars. Geranyl formate is a monoterpene ester synthesized from geranyl and various carboxylic acids, characterized by its distinctive aroma and extensive application in the fragrance, pharmaceutical, and cosmetic industries [23]. Geranic acid has extensive research value and application potential in antibacterial applications, organic synthesis, and biosynthesis [24]. Additionally, fenchone was found to impart a more herbal flavor to ZC than other cultivars. Fenchone is a compound with a pleasant aroma, primarily extracted from sweet fennel essential oil, and is known for its diverse biological activities, including its antioxidant, antibacterial, and cytotoxic properties [25].

Pooling 15 plants per replicate masks within-cultivar variation that may be exploitable for breeding. To assess this variation, we recommend future studies employ single-plant metabolomics coupled with high-throughput platforms (e.g., SPME-GC-MS robotic systems) to capture the full phenotypic spectrum. Volatile profiles are highly sensitive to micro-environmental and seasonal cues [26,27]. Our single-site, single-harvest design limits generalizability. Multi-location trials across contrasting climates and staggered samplings will be required to establish the stability of the observed metabolite signatures before deployment in breeding pipelines. Moreover, this study concentrated solely on the volatile metabolome. Future research that incorporates non-volatile metabolite profiling, such as sugars, organic acids, and amino acids, would yield a more holistic understanding of overall flavor, encompassing taste components. It is also crucial to acknowledge that the associations between chemical profiles and sensory attributes suggested in this study, although grounded in established odor descriptors, are inferential in nature. Future multi-location trials, single-plant resolution metabolomics, and integrated sensory assays are therefore needed to validate and extend these results.

While KEGG pathway enrichment offers valuable preliminary insights, it is crucial to acknowledge that the annotation of plant specialized metabolic pathways, particularly those involving volatile compounds, remains incomplete. Consequently, the enrichment of the monoterpenoid biosynthesis pathway should be interpreted as indicative of a broader metabolic shift rather than as a comprehensive mechanistic map. To definitively elucidate the biosynthetic mechanisms underlying ZC’s superior aroma, the integration of multi-omics data will be essential. Future research will employ RNA-seq and proteomics to identify key genes and enzymes, such as terpene synthases and transferases, that are differentially expressed and responsible for the enhanced production of critical aroma compounds like geranyl formate and fenchone. Establishing correlations between gene expression patterns and metabolite abundance, alongside in vitro validation of enzyme activity, will be vital steps in transitioning from correlation to causation and fully elucidating the genetic basis of this elite flavor phenotype.

In summary, this work demonstrates that coordinated upregulation of terpenoid- and ester-biosynthetic pathways underpins the superior flavor complexity of ZC, furnishing a mechanistic blueprint for flavor-oriented breeding, post-harvest processing optimization, and functional-product development in *M. chinensis* Benth. The integrated analytical pipeline further provides a reference paradigm for volatolomic investigations of other medicinal–edible plant species.

## 4. Materials and Methods

### 4.1. Plant Materials

Four cultivars of *M. chinensis* Benth, specifically from TW, XY, MX, and ZC, were cultivated under uniform agronomic practices in the same experimental field in Wuping County, Fujian Province (Figure 1a) (Figure S4). In August 2023, these four cultivars were harvested from the same location. The aboveground tissues from 15 plants of each variety were collected and combined into a single biological replicate sample. For each species, three identical biological sample replicas were prepared, immediately frozen in liquid nitrogen, and stored at −80 °C for subsequent metabolomics analysis.

### 4.2. Sample Preparation and Extraction

Sample preparation and extraction were performed based on the methods which were provided by the Metware Biotechnology Co., Ltd. (Wuhan, China), exactly as previously described [28,29]. In brief, the samples were cryogenically ground using liquid nitrogen, and approximately 500 mg (1 mL of liquid) of each sample was measured into a headspace vial. A saturated sodium chloride (NaCl) solution and 20 µL of an internal standard solution (at a concentration of 10 µg/mL) were subsequently added to each vial. The sample extraction was then performed using a fully automated headspace solid-phase microextraction (HS-SPME) technique, followed by gas chromatography–mass spectrometry (GC-MS) analysis. The internal standard substance is 3-Hexanone-2,2,4,4-d4.

### 4.3. Chromatography–Mass Spectrometry Acquisition Conditions

At a constant temperature of 60 °C, a 120 μm DVB/CWR/PDMS extraction head was inserted into the headspace vial, and headspace extraction was conducted for 15 min. Subsequently, the analysis was performed at 250 °C for 5 min, followed by GC-MS separation and identification. The separation utilized a DB-5MS capillary column (30 m × 0.25 mm × 0.25 μm, Agilent J&W Scientific, Folsom, CA, USA), with high-purity helium (purity ≥ 99.999%) as the carrier gas at a constant flow rate of 1.2 mL/min. The inlet temperature was maintained at 250 °C, with a splitless injection and a solvent delay of 3.5 min. The temperature program commenced at 40 °C for 3.5 min, increased at a rate of 10 °C/min to 100 °C, then at 7 °C/min to 180 °C, and finally at 25 °C/min to 280 °C, where it was held for 5 min. The electron ionization (EI) source was set at a temperature of 230 °C, with a quadrupole temperature of 150°C and a mass spectrometry interface temperature of 280 °C. The electron energy was 70 eV, and the scanning mode employed was selected ion monitoring (SIM), with qualitative and quantitative ion precision scanning in accordance with food safety national standard [28].

### 4.4. OPLS-DA and PCA Analysis

Agilent MassHunter software, version B.08.00 (Santa Clara, CA, USA) was used to process the peak alignment and integration correction of the data downloaded from the machine. Based on all_sample_data_raw.xlsx, first fill the missing values with 1/5 of the minimum value of each row (metabolite) and then calculate the CV value of the QC sample. Retain substances with CV values less than 0.5 to obtain the final data file all_sample_data.xlsx. (Remove suspicious metabolites with CV values greater than 0.5 in QC samples.) All subsequent analyses were completed based on the data in this table. For OPLS-DA analysis, the data processing method is log_2_ conversion + centralization, and then use R (MetaboAnalystR) (1.0.1) for analysis. For PCA analysis, the data processing method is pareto standardization, and then use the built-in statistical prcomp function of the R software (4.1.2). Set the parameters of the prcomp function as follows: scale = FALSE and center = FALSE.

### 4.5. Differential Metabolite Screening

Compound identification was rigorously performed using a multi-parameter approach to ensure high confidence [29,30]. First, the retention index (RI) of each metabolite was calculated using a homologous series of n-alkanes (C7–C40) and compared with reference values from literature and standard databases. Second, the mass spectrum of each metabolite was matched against standard mass spectral libraries (NIST 14 and Wiley). Furthermore, where available, authentic reference standards were analyzed under identical chromatographic conditions to confirm the identity by matching both retention time and mass spectrum. For accurate quantification, the quantitative ion for each metabolite was selected in strict accordance with the principles of GB 23200.8-2016 and established best practices [31,32]. The selection criteria prioritized, in order, the following: (i) the most abundant fragment ion (base peak) to maximize sensitivity; (ii) a high molecular weight ion with high specificity to the target compound to minimize co-elution interference; and (iii) a stable ion that ensured reproducible integration. The qualitative ions were selected to provide additional confirmation of compound identity. This rigorous process for ion selection was applied to all metabolites, including the internal standard (3-Hexanone-2,2,4,4-d4, quantitative ion: *m*/*z* 62).

Metabolites were preliminarily designated as differential based on their VIP scores derived from the OPLS-DA model, which was built on no fewer than three biological replicates. Features exhibiting a VIP > 1 were retained, as this threshold is widely accepted to indicate a significant contribution to group discrimination. Furthermore, only metabolites displaying an absolute fold change ≥2 or ≤0.5 between the control and experimental groups were considered significantly altered.

### 4.6. Statistical Analysis

For K-means clustering, the metabolite data were first subjected to unit-variance (UV) scaling and then analyzed with the kmeans function in R v4.1.2. Heatmaps were generated from UV-scaled data with the R package ComplexHeatmap (v2.9.4). For KEGG annotation, each metabolite’s C number was mapped to KEGG pathways (http://www.kegg.jp/kegg/pathway.html, accessed on 25 July 2024) and enrichment *p*-values were adjusted using the Bonferroni method for multiple comparisons.

## 5. Conclusions

This study delivers the first comprehensive volatile metabolome map of *M. chinensis* Benth and dissects the molecular basis of the superior aroma signature of the elite ZC cultivar. Through broad-target GC–MS, we confidently annotated 1767 volatiles, revealing terpenoids (19.88%) and esters (18.52%) as the dominant chemotypes. Multivariate statistics (OPLS-DA, K-means) and KEGG enrichment pinpoint monoterpenoid biosynthesis as the central pathway differentiating ZC from three commercial counterparts. Crucially, ZC accumulates >60% of its differential metabolites within terpenoid, ester, and aldehyde classes, translating into an intensified green, sweet, herbal, and fruity sensory profile. Geranyl formate, geranic acid, and fenchone emerge as key odor-active markers, providing quantifiable targets for flavor-oriented breeding and post-harvest processing.

## Data Availability

Data is contained within the article or Supplementary Materials. The data have been deposited in the MetaboLights database, under accession number MTBLS12756, and are publicly accessible at https://www.ebi.ac.uk/metabolights/reviewerb1076910-6880-4864-a9ff-80675b3a0449, accessed on 21 July 2025.

## Supplementary Materials

The following supporting information can be downloaded at: https://www.mdpi.com/article/10.3390/ijms26178713/s1.
