# Supplementary material for: Volatile Flavoromics of Four Mesona chinensis Benth Cultivars: Metabolomic Basis for the Superior Aroma of the Zengcheng Elite Cultivar
Source: Int J Mol Sci. 2025 Sep 7;26(17):8713. doi: 10.3390/ijms26178713 (PMC12428922; doi:10.3390/ijms26178713)
Supplement: Supplementary file 1 [file ijms-26-08713-s001.zip › Supplementary materials-Legend.pdf]

Legend of Supplementary Figures and Table

Figure S1 Metabolic profiles of four *M. chinensis* Benth cultivars determined by GC–MS.

Figure S2 Analysis of volatile component distribution

Figure S3 Supervised OPLS-DA for discrimination among four *M. chinensis* Benth cultivars

Table S1 Identified metabolites, classifications, and relative abundances in four *M. chinensis* Benth cultivars
