# Supplementary figures and images for: Volatile Flavoromics of Four Mesona chinensis Benth Cultivars: Metabolomic Basis for the Superior Aroma of the Zengcheng Elite Cultivar
Source: Int J Mol Sci. 2025 Sep 7;26(17):8713. doi: 10.3390/ijms26178713 (PMC12428922; doi:10.3390/ijms26178713)

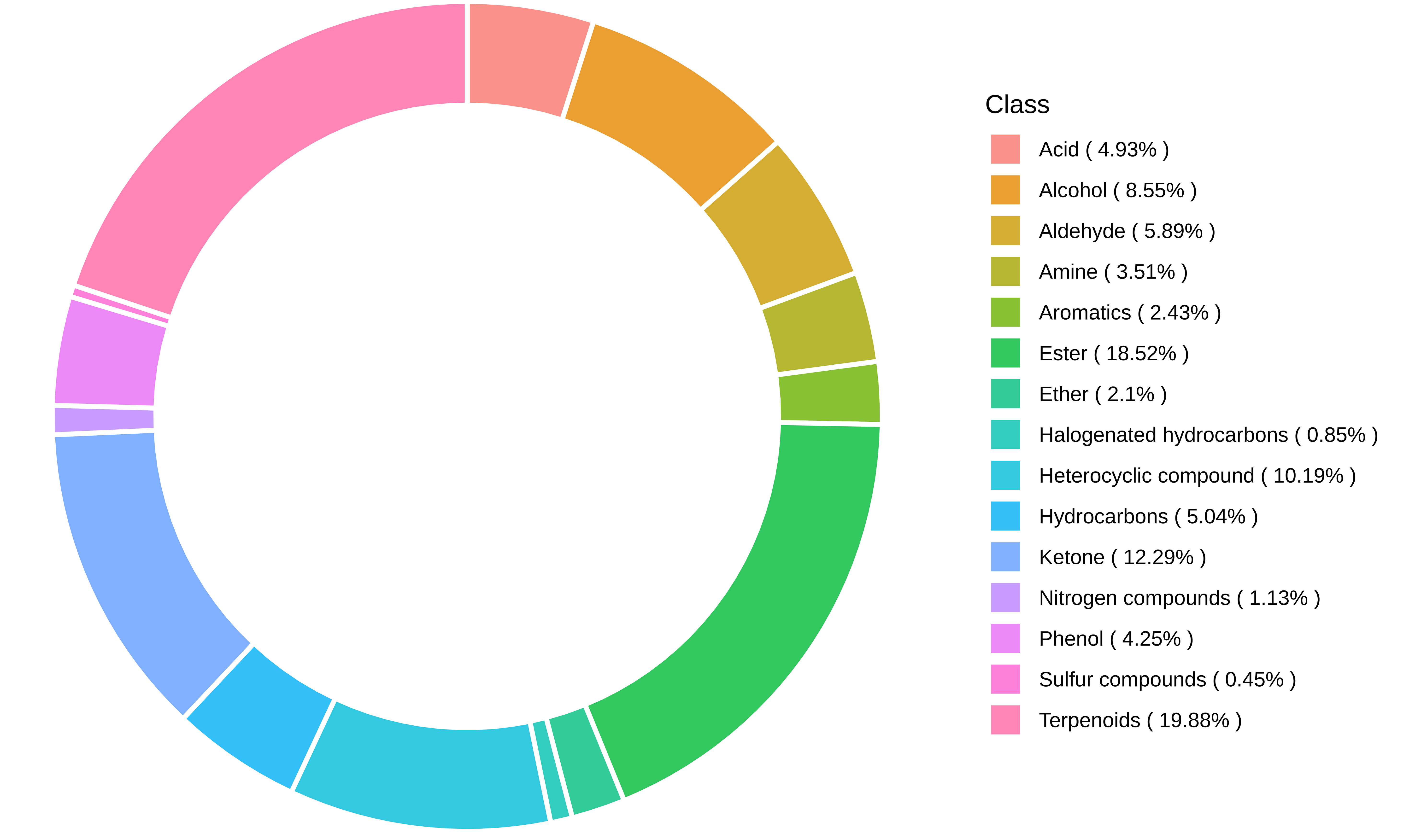

Supplement: Supplementary file 1 [file ijms-26-08713-s001.zip › Figure S1.jpg]

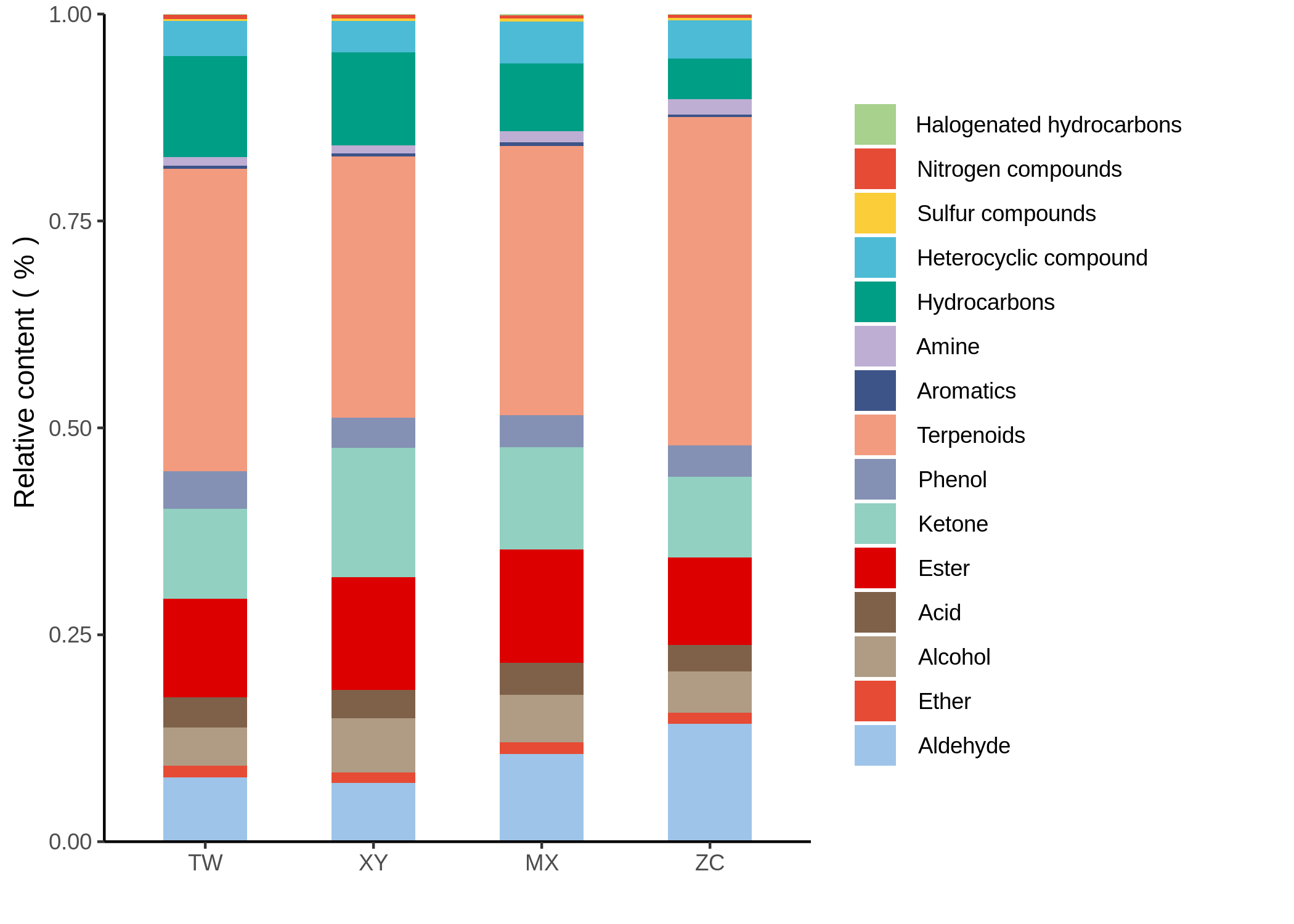

Supplement: Supplementary file 1 [file ijms-26-08713-s001.zip › Figure S2.png]

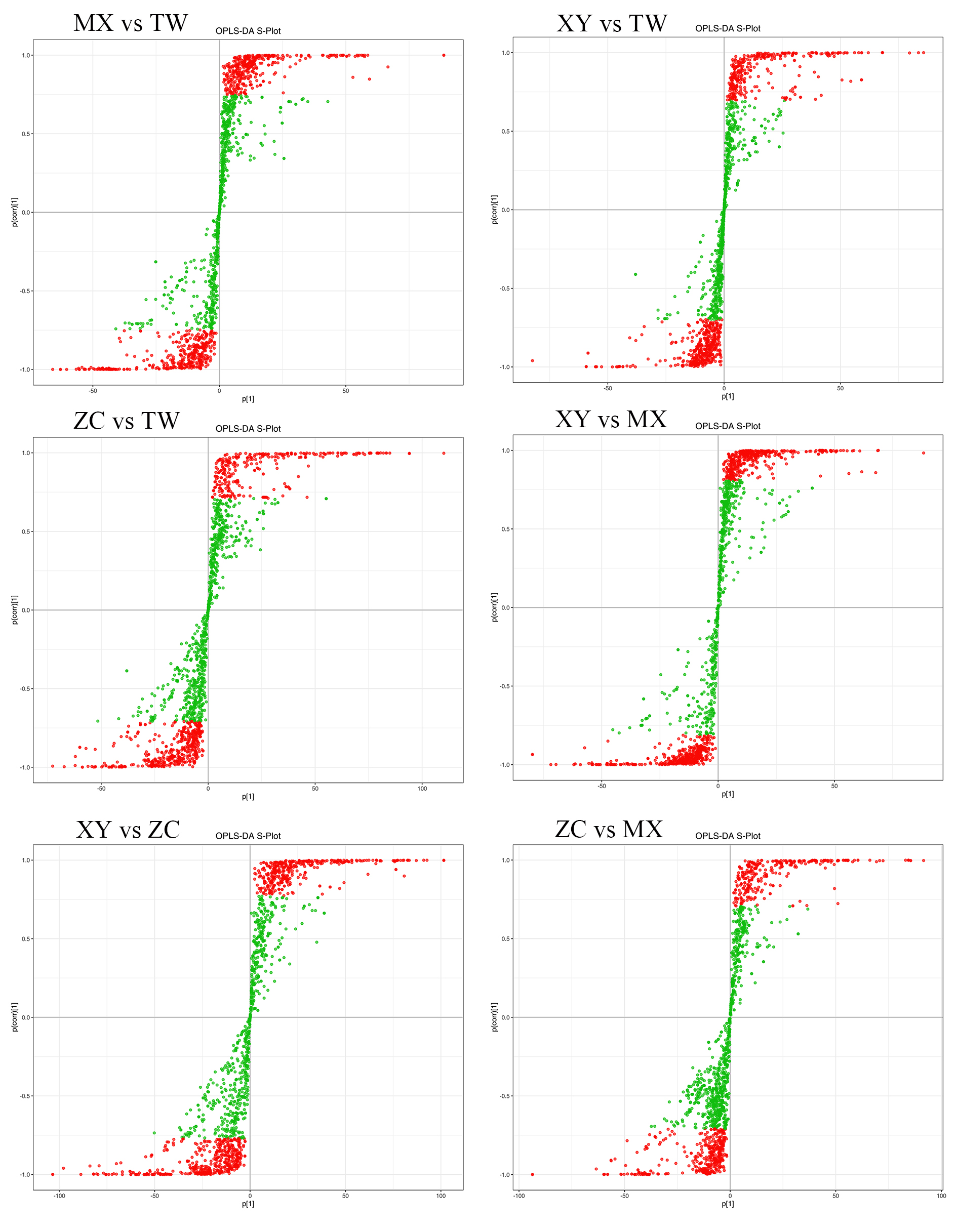

Supplement: Supplementary file 1 [file ijms-26-08713-s001.zip › Figure S3.jpg]

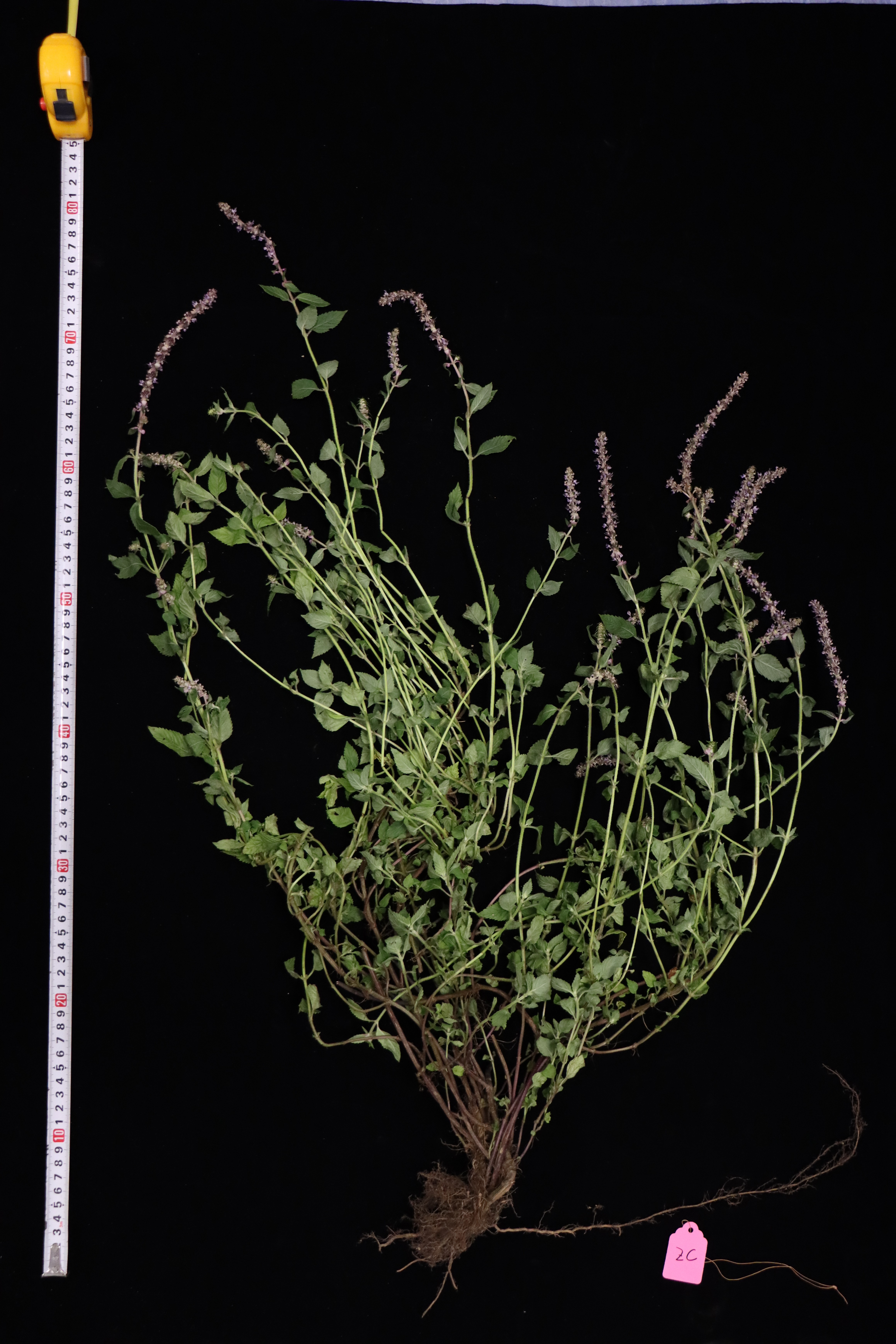

Supplement: Supplementary file 1 [file ijms-26-08713-s001.zip › Figure S4.jpg]
